# New advances in management and treatment of cardiac implantable electronic devices infections

**DOI:** 10.1007/s15010-023-02130-8

**Published:** 2023-11-24

**Authors:** Alessandro Russo, Riccardo Serraino, Francesca Serapide, Enrico Maria Trecarichi, Carlo Torti

**Affiliations:** Infectious and Tropical Disease Unit, Department of Medical and Surgical Sciences, ‘Magna Graecia’ University of Catanzaro, Viale Europa, 88100 Catanzaro, Italy

**Keywords:** Cardiac implantable electronic devices, Infections, Guidelines, PK/PD, Antimicrobial therapy

## Abstract

Cardiac implantable electronic devices (CIED) are increasingly used worldwide, and infection of these devices remains one of the most feared complications.

CIED infections (CDIs) represent a challenge for physicians and the healthcare system in general as they require prolonged hospitalization and antibiotic treatment and are burdened by high mortality and high costs, so management of CDIs must be multidisciplinary.

The exact incidence of CDIs is difficult to define, considering that it is influenced by various factors mainly represented by the implanted device and the type of procedure. Risk factors for CDIs could be divided into three categories: device related, patient related, and procedural related and the etiology is mainly sustained by Gram-positive bacteria; however, other etiologies cannot be underestimated. As a matter of fact, the two cornerstones in the treatment of these infections are device removal and antimicrobial treatment. Finally, therapeutic drug monitoring and PK/PD correlations should be encouraged in all patients with CDIs receiving antibiotic therapy and may result in a better clinical outcome and a reduction in antibiotic resistance and economic costs.

In this narrative review, we look at what is new in the management of these difficult-to-treat infections.

## Introduction

The use of cardiac implantable electronic devices (CIED), including pacemakers (PPMs), implantable cardioverter-defibrillators (ICDs), and cardiac resynchronization therapy (CRT) devices with or without defibrillation capacity (CRT-D or CRT-P, respectively), has increased in recent years worldwide with a concomitant increase of CIED-associated infections [1].

CIED infections (CDIs) present with different clinical scenarios, from pocket infection (PI) to infective endocarditis (IE) and are considered challenging physicians, also considering prolonged hospitalization, prolonged antibiotic therapy, and need of device removal.

Moreover, CIDs are associated with high morbidity and mortality rates as well as a major economic burden on the health care systems [2–4]. Therefore, the management of CDIs must be multidisciplinary by involving different specialists [5].

Aim of this narrative review is to report new advances in management and treatment of CDIs, also exploring the role of new licensed antibiotics and discussing an algorithm about therapeutic management of infections.

## Epidemiology

The exact incidence of CDIs is difficult to define, considering that it is influenced by various factors mainly represented by the implanted device and the type of procedure. As a matter of fact, the incidence of CDIs has certainly changed over time. Data collected and analyzed from Danish nationwide registries including 84,429 patients undergoing CIED surgery from 1996 to 2018 and 1,08,494 CIED operations showed that the CDIs incidence during the device lifetime was considerably higher for ICD and cardiac CRT systems in comparison with PMs; and the risk of reoperations was several folds higher than de novo implantation [6].

A 2018 retrospective analysis of 78,267 French CIED patients followed from 2012 to 2015 reports an infection rate for de novo device implant of 0.5–1.6%. Infection rates were lower for PM (0.5%) and for ICD implants (1.0%) while it was higher for CRT-D and CRT-P (1.6%). Generator replacement procedure, on the other hand, was associated with a higher rate of infection (1.3–3.9%) [7].

Similarly, in a 2019 prospective, multicenter study performed from 2012 to 2016, on 19,599 patients having a CIED procedure, the 12-month infection rate for de novo device implant was 0.3–1.1% compared to an infection rate of 0.5–2.5% for other generator procedures [8]. In addition, some studies showed that new devices without transvenous leads had lower infection rates [9–11]. Specifically, data available suggest that leadless pacemakers have a very low incidence of device-related infection, even when implanted in the presence of active infection. Moreover, as has long been known, the infection rate is highest in the initial period after CIED procedures. [11].

Of importance, several studies indicate that the incidence of CDIs has increased over time. A retrospective analysis on 2,163 US patients from 1988 to 2015 described a trend of increasing incidence of CDIs over the last 2 decades [12]. Similarly in a retrospective single cardiovascular surgery center cohort study of definite CIED infective endocarditis (CIED-IE) episodes between 1981 and 2020, two periods (1981–2000 vs 2001–2020) were compared and CIED- IE was 4.5 times more frequent in the second period, especially in implantable cardiac defibrillators [13]. By contrast, a very recent retrospective analysis on 27,830 Canadian patients (followed for 1 year from 2011 to 2019) showed that patients with implants after 2014 had a decreasing trend in burden of infection, which the authors considered mainly related to enhanced infection prevention and control efforts in Alberta, Canada. [14].

Recently, results from a worldwide survey under the auspices of the European Heart Rhythm Association (EHRA) showed that clinical practices for prevention and management of CIED did not fully comply with current recommendations and demonstrated considerable regional disparities [15]. Finally, there is evidence that reports a seasonal trend in CDIs. Pocket infection incidence (with or without endocarditis) was positively associated with elevated temperature and increased precipitation periods [16].

Then the incidence of CDIs is influenced by several factors, specifically by the type of device with a lower infection rate for PMs and particularly in leadless ones, the type of CIED procedures with a lower infection rate for de novo implants. In addition, several studies showed an increasing trend of CDIs likely due to an increase in the implant of these devices, an increase in age and comorbidities although certainly with a regional disparity. The incidence has also shown a seasonality, but with the advancement of risk recognition and mitigation strategies, an overall CDIs rate of 1% is desirable and achievable [17].

## Risk factors for CDIs

A recent study summarized key information about risk factors for CDIs by analyzing 35 studies. These factors were divided into three categories: device related, patient related, and procedural related, summarized in Table 1 [17]. In the recent analysis of data collected from Danish nationwide registries, risk factors for PI and those for systemic infection were identified.

**Table 1:** CDIs risk factors

**Device**	**Leads and generator**	**Additional interventions**	**Operative approach**
	Two or more leads	Generator replacement	Epicardial
	ICD/CRT	System upgrade	Abdominal device
		Reintervention	
**Patient**	**Underling**	**Transient**	
	Younger age	Recent fever	
	Male	Temporary pacing	
	Renal dysfunction	Anticoagulation	
	Heart disease		
	COPD		
	Immunosuppression		
	Atrial fibrillation		
**Procedural**	**Perioperative**	**Postoperative**	
	Absence of antibiotics	Hematoma	
	Operator inexperience		
	Procedure duration		

*ICD* implantable cardioverter-defibrillator; *CRT* cardiac resynchronization therapy; *COPD* chronic obstructive pulmonary disease

The main risk factors for PI were CIED reoperation (HR 4.66) CRT device (CRT-P HR 1.55 CRT-D HR 2. 12), young age (0–20 HR 2.26), male sex, and prior valvular surgery (HR 1.55, HR 1.62). The main risk factors for systemic infection were conditions that predispose to bacteremia (such as dermatitis), CRT device (CRT-P HR 1.63 CRT-D HR 2.11), young age, CIED reoperations (HR 1.61), male sex, and prior valvular surgery (HR 1.63, HR 2.09). These data also showed that the risk rate is highest in the early post-operative period, especially after CIED reoperations, and hereafter rapidly declining during the first 12 months, until stabilizing at a lower incidence rate [6].

A recent, large cluster crossover trial of conventional *vs* intensive antimicrobial prophylaxis (PADIT) performed in 19,603 patients identified 5 non-modifiable risk factors for CDIs (younger age; procedure type; renal dysfunction; an immunocompromised state; and prior CIED procedures) [18]. In Post hoc analysis of 2,803 control patients from the CDI envelope prophylaxis study WRAP-IT trial, 17 risk factors were identified, of which 5 were non-modifiable: previous procedures (HR 1.03), history of atrial arrhythmia (HR 1.08), device type (CRT-D vs pacemaker/ICD) (HR 1.09), geography (not North America or Europe) (HR 1.30), device type (CRT-P vs pacemaker/ICD) (HR 1.21). Eight risk factors were procedures related (potentially modifiable), length of procedure time, hours (HR 1.09), anticoagulant use at time of procedure (HR 1.08), anticoagulant use (not warfarin or apixaban) (HR 1.17), device implant location (non-left pectoral subcutaneous) (HR 1.10), antiplatelet use at time of procedure (HR 1.15), antiplatelet + anticoagulant use at time of procedure (HR 1.05), complete capsulectomy vs partial or none (HR 1.22), periprocedural use of glycopeptide (vancomycin) vs alternative (primarily cephalosporin) (HR 1.15).

On the other hand, three factors associated with a decrease of the risk of infection were identified: increase in one body mass index unit (HR 0.99), anticoagulant use (apixaban) (HR 0.71), chlorhexidine skin preparation *vs* alternative (primarily povidone-iodine) (HR 0.87), antibiotic pocket wash *vs* non-antibiotic pocket wash or no wash (HR 0.94) [19].

The risk for CIED infection after a device procedure in both PADIT and WRAP-IT was lower than expected and interestingly almost identical; this lower rate of CIED infection, in two of the largest studies conducted in the field to date, underlines that adhering to proper surgical techniques and the use of perioperative antibiotic therapy according to modern era guidelines result in an important reduction in the risk of infection [18, 19].

## Etiology

International consensus document of the EHRA on “how to prevent, diagnose, and treat CDIs” identifies, based on three large patient cohorts in North America, Asia, and Europe, the most frequent etiologies: Gram-positive bacteria (70–80%) especially coagulase-negative staphylococci (CoNS) (37.6% of the isolates) and *Staphylococcus aureus* (30.8%), which is the most common cause of bacteremia. Gram-negative bacteria (GNB) were isolated in 8.9%. Enterobacterales, other Gram- negative rods and fungi were rare [20].

Similarly, a retrospective Spanish single-center cohort study on definite CIED-IE episodes (between 1981 and 2020) identified CoNS as the most frequent etiology of CDIs with an increase in the second period (2001–2020) of methicillin-resistant strain. This etiology was significantly associated with pocket infections and patients with CoNS CDIs showed larger valve vegetation size with significantly more likely removal of the cardiac device system and consequently an increased rate of devices reimplantation. Moreover, an increase of *Enterococcus spp* infections in the second period was identified, probably due to aging and more frequent comorbidities [13].

A single-center, retrospective study analyzed CDIs of 199 French patients from 1992 to 2017 and the major findings were the decline of CoNS, representing 30/50 (60%) of pathogens responsible for CDIs in 1992–1999, 39/86 (45%) in 2000–2008, and 17/63 (27%), in 2009–2017, along with the emergence of *S. aureus* as the primary cause of CDIs during the most recent period (24/63, 38%) [21]. In a recent post hoc analysis of data from PADIT trial, Gram-positive bacteria represented 90% of all reported microorganisms. The most common types of microorganisms were *S. aureus* (35.9%) and CoNS (39.2%) with a low incidence of methicillin-resistant *S. aureus* (MRSA) in patients enrolled mainly from Canadian and Dutch centers. By contrast, a 2016 large single-center US study that included 816 consecutive patients undergoing device removal for confirmed infection showed that patients with CDIs due to MRSA were about 15% [22].

Finally, CDIs caused by atypical pathogens (infection due pathogens rarely or previously not associated with CDIs in humans) were rare but when isolated from blood, tissue, or hardware in patients with CDIs, these pathogens should be considered as etiology of infection and not contaminants considering the crucial role of early diagnosis and targeted treatment. A recent US single-center retrospective analysis of CDIs episodes between 2010 and 2020 found atypical pathogens (i.e., *Corynebacterium striatum* and *Stenotrophomonas maltophilia*) in 5.4% of all CDIs [23].

## What is new in 2023 Duke-ISCVID criteria and in ESC guidelines for infective endocarditis

### 2023 Duke-ISCVID criteria

The Duke Criteria for diagnosis of IE were originally published in 1994 [24] and modified in 2000 [25]. In the very recent consensus document, the International Society for Cardiovascular Infectious Diseases (ISCVID) has modified the latest Duke criteria, and the most important changes relate to CIEDs [26]. First, in the definition of typical organism of the major clinical criteria, the organisms to be considered “typical” pathogens of IE in the presence of intracardiac prosthetic material are also: coagulase-negative staphylococci, *Corynebacterium striatum*; *C. jeikeium, Serratia marcescens, Pseudomonas aeruginosa, Cutibacterium acnes*, non-tuberculous mycobacteria, and *Candida* spp. Second, a Major Criterion regarding imaging and specifically [18F] FDG PET/CT was added and findings for native valve, cardiac device, or prosthetic valve > 3 months after cardiac surgery were considered equivalent to echocardiography. Finally, in the minor criteria, CIEDs implantation was included among the predisposing factors. The main updates are summarized in Table 2.

**Table 2:** Updates to modified Duke criteria proposed by 2023 Duke-ISCVID IE Criteria

Pathologic criteria	
Microorganism identification	Microorganisms identified in appropriate sample by PCR, amplicon or meta-genomic sequencing, or in situ hybridization
Major clinical criteria	
Blood cultures	Removed requirements for timing and separate venipunctures for blood cultures
Definition of typical organisms	Added typical pathogens:
	1) *Staphylococcus lugdunensis*; *Enterococcus faecalis;* all streptococci (S.) except *S. pneumoniae* and *S. pyogenes*; *Granulicatella* spp*.;* *Abiotrophia* spp*.; and Gemella* spp*.*
	2) Organisms to be considered “typical” IE pathogens in the setting of intracardiac prosthetic material: coagulase-negative staphylococci, *Corynebacterium striatum; Corynebacterium jeikeium*, *Serratia marcescens*, *Pseudomonas aeruginosa*, *Cutibacterium acnes*, non-tuberculous mycobacteria, and *Candida* spp.
Other microbiologic tests	Added new major criteria for fastidious pathogens:
	1) PCR or amplicon/meta-genomic sequencing identifies *Coxiella burnetii*, *Bartonella spp*., or *Tropheryma whipplei* from blood; or
	2) IFA > 1:800 for IgG antibodies identifies *Bartonella henselae* or *Bartonella quintana*
Cardiac computerized tomography	Added new Major Criterion. Findings equivalent to echocardiography
[18F] FDG PET/CT	Added new Major Criterion
	Findings for native valve, cardiac device, or prosthetic valve > 3 months after cardiac surgery are equivalent to echocardiography
Surgical	Added new Major Criterion
	Intraoperative inspection constitutes Major Criterion in absence of Major Criterion by cardiac imaging or histopathology
Minor clinical criteria	
Predisposition	Added transcatheter valve implant/ repair, endovascular CIED, and prior diagnosis of IE
Vascular phenomena	Added splenic and cerebral abscess
Immunologic phenomena	Added definition for immune complex mediated glomerulonephritis
Microbiological	Added PCR or amplicon/meta-genomic sequencing evidence of typical pathogen
Imaging	Added PET/CT evidence < 3 months of cardiac surgery
Physical examination	New auscultation of regurgitant murmur when echocardiography is unavailable

*PCR* polymerase chain reaction; *IFA* indirect immunofluorescence assays; *[18F]FDG PET/CT* positron emission computed tomography with 18F-fluorodeoxyglucose; *CIED* cardiac implantable electronic device; *IE* infective endocarditis

### 2023 ESC guidelines for the management of endocarditis

Very recently, new guidelines were developed by the task force on the management of endocarditis of the European Society of Cardiology (ESC). These replace the previous 2015 guidelines by introducing some new features in the management of CDIs [27].

First, following the results of the POET trial, the antibiotic treatment of IE can be divided into two phases. The first phase can last up to 2 weeks of hospital intravenous treatment with combinations of rapidly bactericidal antibiotics. In this initial phase, the device must be removed. After this period, patients who are clinically stable and self-resilient, with a stable home environment, preferably with a cohabitant caregiver self-reliant, may finish antibiotic treatment at home with intravenous (outpatient parenteral antibiotic treatment) or oral antibiotic regimens for up to 6 weeks to eliminate resting bacteria and prevent recurrences. Timing and indications in various clinical scenarios are summarized in Table 3**.**

**Table 3:** Timing and indications in various clinical scenarios to consider outpatient parenteral or outpatient oral antibiotic therapy for infective endocarditis

Critical phase (rapid shift to outpatient parenteral or oral step-down treatment)	Continuation phase (postponed shift to outpatient parenteral or oral step-down treatment)
> 7 days of i.v. antibiotic treatment after non-complicated early lead extraction (< 1 week from admission)	> 2 weeks of i.v. antibiotic treatment after device removal/reimplantation
IE by any causative agent except highly difficult-to-treat microorganisms	Associated right-sided IE with vegetations > 2 cm
No signs of pocket infection	Associated with left-sided IE (apply then criteria for NVE/PVE)
Negative blood cultures at 72 h after reimplantation of CIED	Late or complicated lead extraction
Normal echocardiography	IE by any causative agent except highly difficult-to-treat microorganisms
	No signs of pocket infection
	Negative blood cultures at 72 h after reimplantation of CIED
	Normal echocardiography
	No severe sequelae or clinical complications
	No need for daily and/or complex cures

*CIED* cardiac implantable electronic device; *IE* infective endocarditis; *i.v.* intravenous; *NVE* native valve endocarditis; *PVE* prosthetic valve endocarditis

Another novelty concerns the duration of therapy: in non-*S. aureus* CDIs without valve involvement or lead vegetations, and if follow-up blood cultures are negative without septic emboli, only 2 weeks of antibiotic treatment after device extraction may be considered, while the extension of antibiotic treatment of CDIs to (4–)6 weeks after device extraction should be considered in the presence of septic emboli or prosthetic valves.

## Data about new antibiotics for treatment of CDIS

### Dalbavancin, oritavancin, and telavancin

In a small cohort of 11 early-discharged patients with IE, due to various Gram-positive microorganism, dalbavancin (1.5 g single or twice 1-week apart IV) was shown to be curative in all the cases [28].

In another multicentre, observational, and retrospective study, 83 hospitalized patients with IE and/or bloodstream infection caused by Gram-positive microorganism (34 with IE of whom 23.5% were CIED related) received at least one dose of dalbavancin. The rate of dalbavancin effectiveness to treat IE was 96.7% in a 12-month follow-up period [29].

A good clinical response (92.6%) was observed in another case series of patients with proven IE, of which there were five cardiac device-related IE. However, most of the patients (24 of 27) received also other antibiotics, then it was not clear whether the clinical success was attributable to dalbavancin [30].

In a case report, a patient with recurrent prosthetic valve IE with bacteremia due to vancomycin-resistant *E. faecium* was successfully treated with a prolonged course of oritavancin (10 weeks) in combination with valve replacement surgery [31].

Finally, in an observational study, 151 patients with bacteremia (13 with IE) were treated with telavancin with a positive clinical outcome reported for 74.2% of patients with bacteremia or IE caused by MRSA or other staphylococcus species. [31].

### Ceftaroline and ceftobiprole

Ceftaroline demonstrated potent in vitro activity against a large collection of 23,833 *Staphylococcus aureus* isolates consecutively collected worldwide from patients with BSI, including IE (396) from 2010 to 2019. Ceftaroline was active against 95.2% of IE isolates (MIC_50/90_, 0.25/1 mg/L), with rates of ceftaroline susceptibility higher in North America (99.2%) and Latin America/Asia–Pacific region (LATAM-APAC) (98.3%) than in Europe (92.0%). Among MRSA isolates from IE (*n* = 115; MIC_50/90_, 1/2 mg/L), ceftaroline susceptibility was 98.0% in North America, 90.9% in LATAM-APAC, and 68.5% in Europe [32].

In a retrospective study data, 55 US patients with IE, of which 43.6% were CIED related, caused by Gram-positive bacteria (77.3% caused by MRSA) treated with ceftaroline were analyzed. Clinical success was notably observed in 19 of 23 (82.6%) patients treated with ceftaroline as monotherapy [33].

In a small Italian case series, 12 patients with IE caused by Gram-positive bacteria (including MRSA, methicillin-resistant *Staphylococcus epidermidis*, and methicillin-resistant *Staphylococcus haemolyticus*) were treated with ceftobiprole, 11/12 in combination with daptomycin and 1/12 as monotherapy. In 9/12 (75%) cases, patients were switched to ceftobiprole following failure of previous antimicrobial regimen. In 3/3 patients in which ceftobiprole was administered because of persistently positive blood culture, bacteremia clearance was rapidly achieved. Cure rate was 83.3% (10/12) [34].

### Linezolid and tedizolid

The few data in the literature suggest good efficacy of linezolid in treating IE as monotherapy or in combination with other antimicrobial agents, and treatment of IE with linezolid (LNZ) was not associated with higher mortality rates [35–37]. However, in a recent retrospective study of 292 IE Spanish patients (of whom 57 CDIs), LNZ as definitive treatment of IE was associated with higher in-hospital mortality. Patients were divided into 3 groups based on the therapeutic impact of LNZ: 99 (33.9%) patients in LNZ < 7 days, 11 (3.7%) in LNZ high impact (≥ 7 days, > 50% of the total treatment, and > 50% of the LNZ doses prescribed in the first weeks of treatment), and 178 (61%) in LNZ non-high impact. In-hospital mortality was 51.5%, 54.4%, and 19.1%, respectively. LNZ high-impact patients’ group was characterized by a larger number of comorbidities, more IE complications, and higher frequency of nosocomial acquisition with respect to controls [38].

There are no clinical studies on the use of tedizolid in IE treatment. Some animal models studies showed modest bactericidal activity in vivo and overall lower activity than either vancomycin or daptomycin, while suggesting a possible role for tedizolid in step-down therapy [39, 40].

### New antibiotics for the treatment of multidrug-resistant gram-negative bacteria

No studies have been conducted on the use of the new antibiotics against MDR Gram-negative bacteria for treatment of IEs. A recent case report documented an elderly patient with *P. aeruginosa* XDR (susceptible to only colistin in vitro) who was successfully managed with the addition of cefiderocol (for 4 weeks) to control bacteremia and allow aortic valve replacement [41].

Although Gram-negative bacteria are not a frequent cause of CIED infections and IE in general, the emergence of MDR and extremely resistant Gram-negative pathogens presents a global health challenge and underscores the urgent need for new antibiotics and clinical study to assess their effectiveness [42–44].

In conclusion, studies of new antibiotics especially against Gram-positive bacteria have provided promising data on their efficacy in treating CDIs and IE in general. However, these new antibiotics should not be used as first-line therapy because clinical data are limited. It is necessary to conduct robust clinical trials to provide further information on the pharmacokinetic/pharmacodynamic profile, spectra, in vivo efficacy, and safety of the new drugs before making them available in the clinical practice also for the treatment of CDIs.

The main features and points of interest of the new antibiotics in the treatment of CDIs are summarized in Table 4**.**

**Table 4:** Main features and point of interest of the new antibiotics in the treatment of CDIs

Antibiotics	Class	In vitro activity	Characteristics	Point of interest
Dalbavancin	Semisynthetic lipoglycopeptides	MSSA, MRSA, CoNS, streptococci, VRE vanB	Poor oral bioavailability Half-lives 8.5 days (long acting) Dose adjustment CrCl < 30 ml/min	Promising data for use in IE, but their role need to be validated through clinical trials
Oritavancin		MSSA, MRSA, CoNS, streptococci, VRE Van A-B	Poor oral bioavailability Half-lives 10.3 days (long acting) Not recommended in mild or moderate renal impairment	Dalbavancin and oritavancin are long-acting drugs that have the advantage of being able to reduce the duration of hospitalization
Telavancin		MSSA, MRSA, CoNS, streptococci, VRE vanB	Poor oral bioavailability Half-lives 7–9 h Dose adjustment CrCl < 50 ml/min	Renal dysfunction observed less frequently compared with vancomycin
Ceftaroline	Fifth-generation cephalosporins	MSSA, MRSA, CoNS, streptococci, some *Enterococcus faecalis* isolates	Poor oral bioavailability Half-lives 2.5 h Dose adjustment CrCl < 50 ml/min	Ceftaroline and ceftobiprole are the only cephalosporins active against MRSA Furthermore, although ceftaroline is intrinsically resistant, a synergistic activity with daptomycin has been demonstrated in vitro against VRE [45]
Ceftobiprole		MSSA, MRSA, CoNS, streptococci, some *Enterococcus faecalis* isolates, *P. aeruginosa*	Poor oral bioavailability Half-life 3–4 h Dose adjustment CrCl < 50 ml/min	Therefore, these cephalosporins could be a promising alternative in combination with vancomycin or daptomycin in IE sustained by MRSA, GISA, and VRE Their role need to be validated through clinical trials
Linezolid	Oxazolidinones	MSSA, MRSA, CoNS, streptococci, enterococci including VRE *vanA, vanB*	Optimal oral bioavailability (100%) Half-life 3–7 h No dose adjustmen	Promising data for use in IE, but their role need to be validated through clinical trials. However, the 2023 ESC guidelines suggest the use of linezolid in oral step-down treatment in combination with other antibiotics in the treatment of IE
Tedizolid		MSSA, MRSA, CoNS, streptococci, enterococci including VRE *vanA, vanB*	Optimal oral bioavailability (91%) Half-life ~ 12 h No dose adjustment	Linezolid and tedizolid are the only ones for which an oral formulation with excellent bioavailability is available and do not require adjustment in renal impairment. For linezolid, monitoring is recommended for administration > 14 days due to the risk of myelosuppression
Cefiderocol	Siderophore cephalosporins	Gram-negative	Poor oral availability Half-life 2.8 h Dose adjustment CrCl > 120 ml/min	Cefiderocol is the only one of the new antibiotics active against Gram-negative bacteria for which there are data about its use in IE
			CrCl < 60 ml/min	Although its use in the treatment of IE appears feasible, its use needs to be validated in the future

*MSSA* methicillin-sensible S. aureus; *MRSA* methicillin-resistant S. aureus; *CoNS* coagulase-negative staphylococci; *VRE* vancomycin-resistant enterococci; *CrCl* creatinine clearance; *IE* infective endocarditis; *GISA* glycopeptide intermediately susceptible S. aureus; *ESC* European Society of Cardiology

## Therapeutic management of CDIs

It is now known that the management of IEs and particularly CDIs requires a multidisciplinary approach through the collaboration of multiple specialists, and this reduces the mortality rate of these infections.

The two cornerstones in the treatment of these infections are device removal and antimicrobial treatment. In patients with definite CDIs (systemic and local), complete device removal is recommended (including abandoned leads, epicardial leads, and lead fragments). After diagnosis, the device removal procedure should be performed without unnecessary delay (ideally within 3 days). Antibiotic treatment recommendations as listed in the 2020 EHRA international consensus document and in the 2023 ESC guidelines are summarized in Table 5 and Table 6.

**Table 5:** EHRA 2020 recommended empirical antibiotic therapy regimens according to the clinical scenario

Clinical scenario	Recommended empirical antibiotic therapy regimens
Superficial incisional infection	Flucloxacillin P.O. 1 g every 6–8 h If high MRSA prevalence or penicillin allergy: clindamycin P.O. 450 mg every 6 h, doxycycline P.O. 100 mg every 12 h, and linezolid P.O. 600 mg every 12 h
Isolated CIED pocket infection	Vancomycin i.v. 30–60 mg/kg/day in 2–3 doses Alternative: daptomycin i.v. 8–10 mg/kg every 24 h If systemic symptoms: add ceftriaxone i.v. 2 g every 24 h (or a broader beta-lactam antibiotic) OR gentamycin i.v. 5–7 mg/kg every 24 h
CIED systemic infection (including suspicious positive blood cultures in a patient with a CIED)	Vancomycin i.v. 30–60 mg/kg/day in 2–3 doses Alternative: daptomycin i.v. 8–10 mg/kg every 24 h + Ceftriaxone i.v. 2 g every 24 h (or a broader beta-lactam antibiotic) OR gentamycin i.v. 5–7 mg/kg every 24 h If staphylococcal prosthetic valve infection: add rifampicin P.O. or i.v. 900–1200 mg/day in 2 doses after 5–7 days

*CIED* cardiac implantable electronic device; *MRSA* methicillin-resistant S. aureus; *P.O.* per os; *i.v.*: intravenous

**Table 6:** 2023 ESC guidelines recommendations for antibiotic treatment of CIED infective endocarditis due to MSSA, MRSA, *Enterococcus* spp., and VRE

Pathogen	Recommended antibiotic therapy regimens
MSSA	(Flu)cloxacillin 12 g/day i.v. in 4–6 doses Cefazolin 6 g/day i.v. in 3 doses (only in patients with non–immediate-type hypersensitivity reactions to penicillin) **Allergy to beta-lactam** Cefazolin 6 g/day i.v. in 3 doses Daptomycin 10 mg/kg/day i.v. in 1 dose (may be considered) ** + ** Ceftaroline 1800 mg/day i.v. in 3 doses OR Fosfomycin 8–12 g/day i.v. in 4 doses
MRSA	Vancomycin 30–60 mg/kg/day i.v. in 2–3 doses Daptomycin 10 mg/kg/day i.v. in 1 dose (may be considered) ** + ** Cloxacillin 2 g/day i.v. in 6 doses OR Ceftaroline 1800 mg/day i.v. in 3 doses OR Fosfomycin 8–12 g/day i.v. in 4 doses
*Enterococcus spp.*	Amoxicillin 200 mg/kg/day i.v. in 4–6 doses Ampicillin 12 g/day i.v. in 4–6 doses Ceftriaxone 4 g/day i.v. in 2 doses Gentamicin 3 mg/kg/day i.v. or i.m. in 1 dose
Beta-lactam resistant Enterococcus spp. (*E. faecium*)	Vancomycin 30 mg/kg/day i.v. in 2 doses Gentamicin 3 mg/kg/day i.v. or i.m. in 1 dose
VRE	Daptomycin 10–12 mg/kg/day i.v. in 1 dose Ampicillin 300 mg/kg/day i.v. in 4–6 equally divided doses Fosfomycin 12 g/day i.v. in 4 doses Ceftaroline 1800 mg/day i.v. in 3 doses Ertapenem 2 g/day i.v. or i.m. in 1 dose

*MSSA* methicillin-sensible S. aureus; *MRSA* methicillin-resistant S. aureus; *VRE* vancomycin-resistant enterococci; *i.v.* intravenous

A very important key point in the management of CDIs is the reimplantation of the device for which the indication for reimplantation must always be carefully evaluated. Currently, there are insufficient data regarding the timing of reimplantation which according to the 2023 ESC guidelines should be performed at a site distant from the previous generator site and should be delayed until signs and symptoms of local and systemic infection have resolved and blood cultures are negative for at least 72 h after extraction in the absence of vegetations or fibrous remnants or after 2 weeks of negative blood cultures if vegetations have been visualized.

For patients at high risk of sudden cardiac death and for pacemaker-dependent patients, it seems reasonable to use temporary devices until symptoms and signs of systemic infection have resolved before implanting a permanent device. Alternative devices, such as leadless pacemakers or subcutaneous ICDs, which available data suggest have a low infection rate, may be implanted in selected patients if the risk of new infections is considered high [9, 10, 27].

**Fig. 1:**
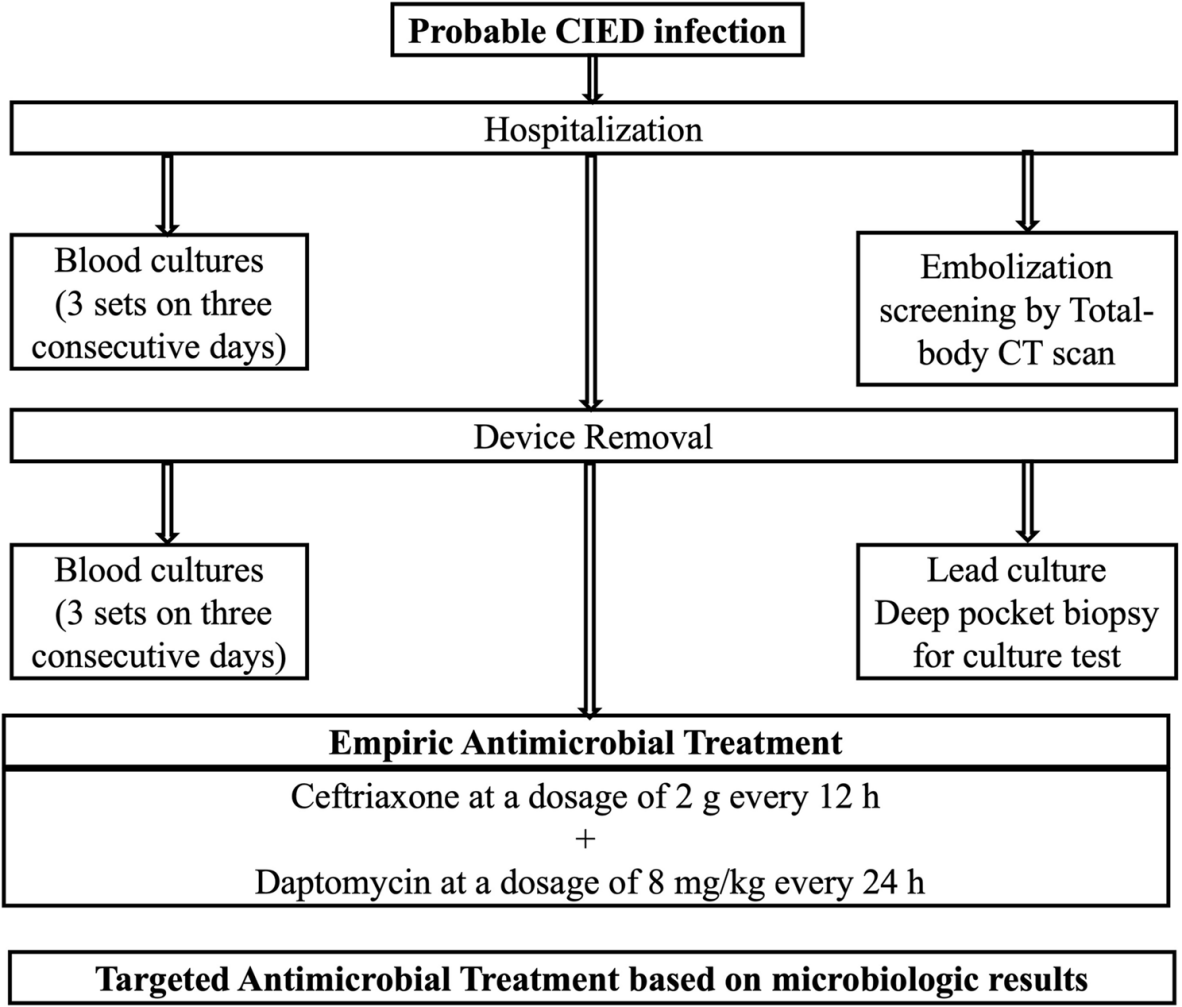
Therapeutic management of empiric therapy of CDIs with vegetation on leads and/or valves

In Fig. 1**,** we report the proposed algorithm to improve microbiological diagnosis and to manage empiric therapy of CDIs with vegetation on leads and/or valves, with or without embolism, and with or without pocket infection.

As a matter of fact, vancomycin has been long recommended as the treatment of choice for staphylococcal isolates, especially methicillin-resistant strains [46]. Many studies demonstrated that mortality associated with *Staphylococcus aureus* bacteremia (SAB) was significantly higher when the empirical antibiotic is inappropriate and when vancomycin is empirically used for treatment of infection with strains with a vancomycin MIC > 1 μg/mL [47]. Of interest, meta-analyses found a correlation between higher vancomycin MICs and unfavorable outcome [48–50]; conversely, another meta-analysis did not find statistically significant differences about mortality when comparing patients with *S aureus* strains with a vancomycin MIC ≥ 1.5 μg/ml to those with low-vancomycin MIC (< 1.5 μg/ml) [51]. Of importance, outcomes of patients with SAB are also related to various clinical confounding factors such as source control (e.g., removal of infected vascular catheters, drainage of abscesses) and underlying diseases, which may bias the results of these studies. Thus, although a definite conclusion cannot be reached, vancomycin should be considered a second-choice drug in patients with infecting MRSA strains having MIC > 1 μg/ml.

Teicoplanin resulted to be clearly less efficacious than antistaphylococcal penicillins and vancomycin in cases of intravascular staphylococcal infections [52, 53]. In addition, many reports have demonstrated the emergence of coagulase-negative staphylococcal species, especially *Staphylococcus haemolyticus*, expressing heteroresistance or full resistance to this drug [54].

Of importance, during the last years, new drugs active against MRSA have been introduced. Out of these, the role of daptomycin is increasing also considering that it in a randomized trial was compared with vancomycin for patients with SAB and was not inferior to standard therapy [55].

Daptomycin may be considered as a first-line therapy in intravascular infection caused by staphylococcal strains [56]. High-dose daptomycin (8–10 mg/kg once daily) in combination with other antibiotics has been recommended for persistent MRSA bacteremia when isolates are susceptible to daptomycin or when organisms have a high vancomycin MIC (e.g., > 1 µg/mL) [57–59]. In a prospective cohort study of patients with left-sided IE, high-dose daptomycin was not significantly associated with any difference for in-hospital mortality compared with standard of care [60], and these data were confirmed also in other studies [61]

Some recent articles have evaluated the efficacy of daptomycin combined with other β-lactams for the treatments of patients with staphylococcal infections, including biofilm-associated infections. Daptomycin plus β-lactams (including nafcillin, cefotaxime, amoxicillin clavulanate, and imipenem) showed to be highly synergistic against both heterogeneous and homogeneous clinical MRSA strains. As a matter of fact, β-lactams induced a reduction in the cell net positive surface charge, an effect that may favor the binding of daptomycin to the cell surface; importantly, the combination of daptomycin and a β-lactam prevented the selection of daptomycin-resistant variants [62]. Clinical experiences showed efficacy of combination of daptomycin and β-lactams in treating persistent MRSA bacteremia [63], and a multicentre study confirmed that the overall treatment efficacy of daptomycin was enhanced after the addition of a β-lactam in patients with MRSA bacteremia associated with IE or bacteremia from an unknown source [64]. An important additive effect has been demonstrated for ceftaroline [65]: daptomycin plus ceftaroline was used in 26 cases of persisting staphylococcal bacteremia (20 MRSA, 2 VISA, 2 MSSA, 2 methicillin-resistant *S epidermidis*); after daptomycin plus ceftaroline was started, the median time to bacteremia clearance was 2 days (range, 1–6 days) with recovery of patients [66].

Of interest, a progressive increase in enterococcal CDIs has been described over the last 3 decades [67], and it is of special interest owing to its severity and therapeutic difficulties due to an increasing rate of antimicrobial resistance. As reported above, *Enterococcus faecalis* is the leading species causing BSI or IE, and accounts for about the 65–70% of the cases while *E. faecium* for about the 25%. In USA, approximately 12% of the hospital-acquired infections are *Enterococcus* species.

Enterococci are relatively resistant to the killing effects of cell wall–active agents (penicillin, ampicillin, and vancomycin) and are impermeable to aminoglycosides. Therefore, a combination regimen of two agents, a cell wall–active agent with a synergistically active aminoglycoside is required for optimal cure rates of invasive infections, such as BSI or IE. Combination of ampicillin plus gentamicin has been long considered the regimen of choice, but during last 2 decades, further combinations have been tested. Combination of ampicillin and ceftriaxone may saturate low-molecular-weight penicillin-binding proteins (PBPs) 2, 3, 4 and 5, producing the bactericidal synergistic effect [68, 69]. Since enterococcal endocarditis appears generally in older patients, and age is associated with a higher risk of nephrotoxicity, less-toxic regimens like ampicillin plus ceftriaxone may be preferred. Important data were recently published about the role of ampicillin and ceftriaxone combination in the treatment of *Enterococcus faecalis* infective endocarditis (EFIE). In an observational, nonrandomized, comparative multicenter cohort study, the ampicillin–ceftriaxone combination was as effective as ampicillin plus gentamicin for treating *E. faecalis* infective endocarditis [70]. Ampicillin–ceftriaxone combination was effective in both high‐level aminoglycoside resistance and non-high‐level aminoglycoside resistance EFIE.

VRE infections have been associated with adverse outcomes. The magnitude of this effect was illustrated in a meta-analysis of 9 studies of 1614 enterococcal bloodstream infections, 42 percent of which were due to VRE [71]. The mortality rate was significantly higher in patients with VRE compared with vancomycin-susceptible enterococcal isolates; however, it is difficult to ascertain the exact role of VRE infection to determine death because these organisms frequently colonize or infect compromised patients with severe underlying diseases. An antimicrobial therapy is recommended in patients with at least two or more positive blood cultures associated, or a single positive blood culture accompanied by signs of sepsis. Daptomycin and linezolid are feasible options in cases of VRE infections. A recent meta-analysis shows that linezolid treatment for VRE bacteremia was associated with a lower mortality than daptomycin treatment [72].

### Pharmacokinetic/pharmacodynamic (PK/PD) considerations in the management of CDIs

Critically ill patients with CDIs and a concomitant BSI can show several dysfunctions related to the septic syndrome which, together with drug interactions and other therapeutic interventions (e.g., inotropes and continuous renal replacement therapies), may affect drug pharmacokinetics [73]. Variations in the extracellular fluid content and/or in renal or liver function are the most relevant and frequent pathophysiological mechanisms possibly affecting drug disposition in critically ill patients; hydrophilic antimicrobials (e.g., β-lactams, aminoglycosides, and glycopeptides) and renally excreted, moderately lipophilic, antimicrobials (e.g., ciprofloxacin, gatifloxacin, and levofloxacin) have to be considered at high risk of presenting substantial daily fluctuations in plasma concentration during.

Under these circumstances, higher dosages for most hydrophilic antimicrobials (either aminoglycosides or β-lactams) should, therefore, be considered to ensure therapeutic concentrations are maintained, and therapeutic drug monitoring (TDM) may be of great value in the clinical conditions described above.

Pharmacokinetics of vancomycin shows broad variability in critically ill patients due to a significant change in both clearance and the Vd [74]. Higher doses of vancomycin seem to be necessary in critical patients, even when the pathogens have MIC values typical of susceptible microorganisms, and TDM is strongly recommended. According to a PK/PD analysis, vancomycin standard dosages lead to a 33% risk of not achieving the recommended AUC_0–24_/MIC breakpoint for *S. aureus* in ICU patients, possibly leading to an unfavorable clinical outcome [75]. The results of Monte Carlo simulation revealed that doses of 3000 mg or even 4000 mg daily may be necessary to reach the highest probability of efficacy when susceptible *S. aureus* strains are involved in critically ill patients, and similar results were found for other staphylococcal isolates. With the aim of improving the results of vancomycin therapy, a variety of strategies such as higher doses, combination therapy, and continuous infusion have been proposed. Continuous infusion might make treatment monitoring and adjustment easier and cheaper because vancomycin concentrations in serum are less variable and more sustained [76]. In a prospective multicentre randomized trial comparing critically ill patients with severe methicillin-resistant staphylococcal infections, continuous infusion of vancomycin resulted in therapeutic concentrations being achieved more quickly, less AUC variability between patients, fewer samples required to monitor treatment, and reduced 10-day antibiotic cost; clinical efficacy and safety were comparable to the intermittent infusion schedule [77]. In an important study, authors observed more favorable clinical outcomes in patients with continuous infusion of vancomycin in terms of improved organ function and leukocyte response [78]. The evidence suggests a strict monitoring of vancomycin serum concentrations in critically ill patients and the preference for continuous infusion at least in strains fully susceptible (MIC < 1 μg/ml).

With regard to daptomycin, Safdar et al. demonstrated that both the AUC/MIC_0–24_ ratio and the C_max_/MIC ratio were strong predictors of in vivo efficacy [79, 80] of the drug. Using an in vitro pharmacodynamic model with simulated endocardial vegetations, Cha et al. compared daptomycin at 6 and 8 mg/kg/day vs vancomycin at 1 g every 12 h against MRSA, methicillin-resistant *Staphylococcus epidermidis*, glycopeptide-intermediate *S. epidermidis*, and VRE [81]. Both daptomycin regimens achieved greater killing (more than 99.9% kill by 8 h) and greater bacterial reduction than vancomycin against all tested isolates at 24, 48, and 72 h. A further clinical experience showed that patients with MRSA BSI and severe sepsis or septic shock may experience a significant reduction of daptomycin serum levels, leading to lower exposure and poor clinical outcome [82]. The underexposure of daptomycin was related to an increased clearance of the drug and was independent from weight and from the dosage used since it was detected also in patients receiving 8 mg/kg/day. Monte Carlo simulations showed that a fixed dosage of 750 mg/die might be the best choice to optimize the drug exposure and to minimize side effects in septic patients [83]. A simple method to calculate daptomycin AUC may also be used to adjust dosages in the clinical practice [84]. These findings suggest that higher daptomycin doses are likely necessary at the onset of therapy in critically ill patients, and that future interventional randomized studies are needed to clarify the best daptomycin dosing [85, 86].

TDM and PK/PD correlations should be encouraged in all patients with BSI or IE receiving antibiotic therapy and may result in a better clinical outcome and a reduction in antibiotic resistance and economic costs.

## Conclusion

In conclusion, CDIs represent a major problem burdened by high morbidity and mortality and a major expense for healthcare systems. The management of these infections is very complex and must be handled by experienced personnel. Over the last 20 years, new antibiotics have shown promising results, but given the limited clinical data, their use should be limited to specific cases, pending further clinical studies providing more information on the PK/PD profile, in vivo efficacy, and safety of these new drugs. However, the increasing prevalence of multidrug-resistant pathogens makes this necessary as a matter of urgency.

## Data Availability

Not applicable.
